# Effect of an Early Oral Food Intake Strategy on the Quality of Life of Postoperative Patients With Esophageal Cancer

**DOI:** 10.3389/fsurg.2022.872221

**Published:** 2022-06-16

**Authors:** Renmei Yang, Wenxiu Yuan, Zhengfang Li, Manrong Yang, Yuequan Jiang

**Affiliations:** Department of Thoracic Tumor Center, Chongqing University Cancer Hospital, Chongqing, China

**Keywords:** accelerated recovery, early food intake, quality of life, endoscopic surgery, esophageal cancer

## Abstract

**Objective:**

To explore the early oral food intake on the quality of life of postoperative patients with esophageal cancer.

**Methods:**

A total of 100 patients with esophageal cancer were randomized into an observation group and a control group, with 50 patients in each group. The patients in the control group were routinely indwelt with a gastric tube and fasted for seven days. If no abnormality was found in examinations, the patients were instructed to attempt drinking water and gradually try eating liquid, semi-liquid, and common foods. The patients in the observation group were subjected to the early oral food intake strategy. The recovery and gastrointestinal symptoms of the patients were evaluated using the six-minute walk test and gastrointestinal symptom rating scale (GSRS) at discharge. The quality of life of patients was evaluated using the QLQ-C30 scale and QLQ-OES18 scale during the return visit to the hospital one month after discharge.

**Results:**

The GSRS score of the observation group was markedly lower than that of the control group. The six-minute walk distance in the observation group was significantly higher than that in the control group; the difference was statistically significant (*P* < 0.01). In comparing the QLQ-C30 scores of the two groups, the scores in physical function, emotional function, and general health condition in the observation group were higher than those in the control group. In comparing the QLQ-OES18 scores of the two groups, the scores in dysphagia, eating, reflux, pain domains, and choking symptoms in the observation group were lower than those in the control group; the differences were statistically significant (*P* < 0.01), and there were no statistically significant differences in other symptoms and related functions between the two groups (*P* > 0.05).

**Conclusion:**

The early oral food intake strategy can reduce gastrointestinal symptoms, promote recovery of postoperative patients with esophageal cancer, and improve quality of life.

## Introduction

Esophageal cancer is a common gastrointestinal tumor, and the disease takes the eighth position in incidence and sixth position in mortality around the world. Surgical treatment is still the first choice for resectable esophageal cancer (1). Surgery methods include minimally invasive radical resection of esophageal cancer and traditional open radical resection of esophageal cancer (2). Regardless of a minimally invasive surgery or open surgery option, one-half to one-third of the distal stomach is usually resected during surgery and used as the major reconstructed organ of the digestive tract–tubular stomach after esophagectomy (3). The original physiological, anatomic structure of the tubular stomach changes, and patients tend to present gastrointestinal symptoms such as nausea, vomiting, and reflux of gastric contents after food intake, which increases the physical and psychological burden of food intake (4) and results in malnutrition, eventually decreasing quality of life (5). Therefore, for postoperative patients with esophageal cancer, alleviating gastrointestinal symptoms, ensuring successful food and nutrition intake, accelerating recovery, and improving quality of life is the current focus requiring close attention. Studies have demonstrated that early oral food intake after minimally invasive esophageal cancer surgery is safe and feasible (6). Therefore, this paper aims to use the early oral food intake to help postoperative patients with esophageal cancer adapt to the changes in digestive tract mode as early as possible, alleviate gastrointestinal symptoms, promote rapid recovery, and improve quality of life.

## Materials and Methods

### Subjects

A total of 100 patients subjected to esophageal cancer surgery in our department from January 2020 to December 2020 were selected. The patients were randomized into an observation group and a control group, with 50 patients in each group. There were no significant differences in gender, age, disease stage, and nutritional status between the two groups (all *P* > 0.05). All patients were subjected to the same surgery by the same surgical team.

The inclusion criteria were as follows: ① patients were first diagnosed with esophageal cancer and could receive laparothoracoscopy combined with radical dissection of esophageal cancer; ② preoperative organ function examinations indicated a tolerance to surgery and the Karnofsky performance score (KPS) score was ≥80; ③ no history of serious cardiopulmonary disease; ④ patients were transferred to the general ward directly after surgery or transferred to the general ward from ICU within one day after surgery; ⑤ patients signed an informed consent and voluntarily participated in this study.

The exclusion criteria were as follows: ① unsatisfactory intraoperative anastomosis; ② patients could not be transferred back to the ward on schedule due to condition changes; ③ patients had bleeding in the stomach after surgery, or had daily coffee-colored fluid drainage from the gastrointestinal decompression tube, with a drainage volume of >200 mL; ④ patients did not follow the food intake scheme after surgery.

None of the recruited patients had neoadjuvant therapy. All of them were newly diagnosed with esophageal cancer.

### Specific Measures

The surgical method used was a laparothoracoscopy combined with radical resection of esophageal cancer. Anastomosis at the esophagogastric neck was performed using the Jiang’s anastomosis technique (7), and the tip of gastric tube was adjusted to below the anastomosis during surgery.

The eating strategy management team included 1 chief nurse, 2 attending physicians in the Department of Thoracic Surgery, and 4 responsible nurses. The strategies were formulated by the team members. The attending physician evaluated patients’ eating and guided the type and amount of daily eating. The responsible nurses were responsible for supervising the implementation, observing and evaluating the effect, and also instructing the family members to prepare food according to the patient’s eating requirements and amount. The chief nurse performed quality control of the entire process.

In the observation group, specially assigned persons explained the advantages and details of early oral food intake to the patients and their families.

After specific surgery, the patients’ vital signs were stable after recovery from anesthesia. Specially assigned persons evaluated the patients’ understanding of the early oral food intake scheme, and the unmastered parts were emphasized and supplemented on that day. The patients had no bleeding in the stomach within 24 h after surgery, and the drainage volume of the gastrointestinal decompression tube was <200 mL. The patients were instructed to start oral food intake 24 h after surgery, and the gastric tube was continuously clamped. The feeding sequence was as follows: clear fluid (5% glucose and sodium chloride injection), liquid (milk, rice water, etc.), semi-liquid (mainly thin gruel), and common food. Clear liquid, namely oral 5% glucose and sodium chloride injection, was fed five to six times per day at 38°C–40°C and 10–30 mL per time, and the intake was controlled within 200 mL per day. The patients had no discomfort after feeding. In 48 h after surgery, the gastrointestinal decompression tube was opened for 30 min to observe the drainage volume. When the drainage volume was less than 200 mL, the gastric tube was removed according to the medical instructions and the patients were fed mainly liquid food, such as soups and fruit juice, for six to eight times per day and at 500–1,000 mL per day. The patients had appropriately increased intake, with the applied principle of less volume to more volume and thin liquid to thick liquid by the third day after surgery. On the fourth day after surgery, the patients were fed semi-liquid food, such as a steamed egg, congee, and soft noodles, six times at 200 mL per time. If the intake was gradually increased, the frequency of intake was decreased appropriately. After each feeding, the patients were instructed to carry out bedside activities, indoor activities, and in-ward activities using the rehabilitation walking aids, and the activity amount gradually increased with their physical recovery. If no discomfort occurred after feeding three to four weeks after surgery, the intake of soft food or solid food was appropriately allowed. It was recommended to take 25–50 g of steamed bread without water every day for three to six months in order to prevent anastomotic stenosis. The nutrition requirements not met by oral food intake were supplemented via peripheral venous infusion during hospitalization. The intravenous infusion volume gradually decreased day by day according to food intake. The family members and patients jointly listened to the instructions on each food intake. The food intake was quantified accurately and refined, and detailed instructions with pictures, videos, and other materials were provided. The patients were closely observed every day for nausea, vomiting, abdominal pain, abdominal distension, diarrhea, and other gastrointestinal symptoms. Instructed on each intake, the patients mastered and adapted to a food type, and then attempted the next type of food. The explanation for fine food intake instructions and data collection were carried out by four nurses in charge that had received homogenized training. The follow-up period was one month after discharge.

In the control group, the patients were routinely indwelt with a gastric tube and they fasted for five to six days after surgery. The patients received parenteral nutrition according to medical instructions during fasting, that is, they were infused with Kabiven and other nutrients via central veins. On the seventh day after surgery, the patients were subjected to an upper gastrointestinal tract angiography with meglumine diatrizoate according to medical instructions to observe the obstruction of anastomosis and pylorus emptying. If no abnormality was found, the gastric tube was withdrawn according to medical instructions, and the patients were instructed to observe for nausea, vomiting, abdominal pain, abdominal distension, diarrhea, and other discomforts, as well as instructed to attempt drinking water. In case of no discomfort, the patients were instructed to gradually introduce food from the next stage and to follow the principle of eating multiple meals with less food each meal, eating thin food to dry food, and carefully chewing and swallowing food slowly.

The instructions of new food to patients in different groups are shown in Table 1.

**Table 1:** Instructions of new food to patients in different groups.

Groups	Time point	Food
Observational group	24 h after surgery	Clear liquid; 5–6 times per day, 10–30 mL per time; <200 mL per day
	48 h after surgery	Liquid food; 6–8 times per day, 500–1,000 mL per day
	4th day after surgery	Semi-liquid food; 6 times per day, 200 mL per time
	3–4 weeks after surgery	Soft food or solid food; 25–50 g of steamed bread without water every day for 3–6 months
Control group	5–6 days after surgery	Fasted
	7th day after surgery	Start food intake: multiple meals with less food in each meal

### Evaluation Indices

① The gastrointestinal symptoms were evaluated by the gastrointestinal symptom rating scale at discharge after surgery. Su et al. (8) in China used this scale to self-evaluate the incidence and severity of gastrointestinal symptoms in patients in the last two weeks. The scale included 18 items and six dimensions: abdominal pain (including three items), reflux (two items), dyspepsia (four items), diarrhea (three items), constipation (three items), and eating disorders (three items). The severity of each symptom was expressed by the 7-point Likert score as follows: 0 = normal; 1 = seldom; 2 = a little; 3 = moderate; 4 = slightly severe; 5 = severe; 6 = very severe. A dimension score was calculated by averaging the sum of the scores in each dimension, with a maximum score of 6 and a minimum score of 0. The total confidence and sub-dimension confidence of the scale ranged from 0.43 to 0.87. ② The recovery of the patients was evaluated using the six-minute walk test. The six-minute walk test was conducted according to its operation guidelines (9).

The quality of life of the patients was evaluated using the QLQ-C30 scale (10) and the QLQ-OES18 scale (11) during the return visit to the hospital one month after surgery.

The QLQ-C30 scale had 30 items, including five functional domains (physical function, role function, emotional function, social function, and cognitive function), three symptom domains (fatigue, pain, nausea, and vomiting), and one general health condition/six single items in the quality of life domain.

The special scale for esophageal cancer (EORTC QLQ-OES18) is a subscale of EORTC QLQ-C30. Their combination was used to evaluate the quality of life of patients with esophageal cancer. The scale included 18 items for determining esophageal cancer-related symptoms and treatment side effects, which was divided into four domains (dysphagia, eating, reflux, and pain) and six symptom items (trouble with saliva, choking, dry mouth, taste, cough, and speech), each as a domain.

The original scores of QLQ-C30 and QLQ-OES18 were converted into 0–100 by a linear formula. A higher score in the functional scale indicated a better functional status, a higher score in general health condition indicated a better quality of life, and a higher score in the symptom scale and single items indicated more significant symptoms (12).

### Statistical Treatment

The SPSS26.0 software was used for statistical description. The measurement data were expressed by mean ± standard deviation (SD), and the enumeration data were expressed by frequency (%).

For baseline and effect comparison, the enumeration data were analyzed using the chi-square test (*χ*^2^). The measurement data were analyzed using the normality test and homogeneity test for variance. If there was normal distribution and homogeneous variance, independent samples were used for the t-test. If the data violated normal distribution and homogeneity of variance, the rank-sum test was used. For abnormal distribution, the Wilcoxon signed-rank test in non-parametric tests was used. A *P* value of <0.05 indicated a statistically significant difference.

## Results

The baseline characteristics of patients in different groups are summarized in Table 2. There was no significant different in gender, age, educational level, body mass index, complication, lesion location, pathological stage, duration of surgery, and bleeding volume during surgery between the observational and control groups (Table 2). Moreover, no significant difference was observed in the incidence of anastomotic leakage, anastomotic stenosis, delayed gastric emptying, and pulmonary infection between the two groups (Table 3).

**Table 2:** Baseline characteristics of patients in different groups.

Parameters	Observational group (*n* = 50)	Control group (*n* = 50)	*χ*^2^/*t*	*P*
Gender			0.667	0.414
Male	32	28		
Female	18	22		
Age (years, mean ± SD)	60.62 ± 6.74	60.28 ± 6.61	0.255	0.799
Educational level			1.674	0.643
Elementary school	8	5		
Junior high school	21	26		
High school	13	10		
College and above	8	9		
Body mass index (kg/m^2^, mean ± SD)	22.42 ± 1.30	21.97 ± 1.25	1.763	0.081
Complications			0.796	0.372
Hypertension	3	2		
Diabetes	3	5		
Lesion location			1.681	0.432
Upper	8	10		
Middle	30	33		
Lower	12	7		
Pathological stage			0.460	0.795
Stage I	12	15		
Stage II	28	26		
Stage III	10	9		
Duration of surgery (min, mean ± SD)	198.32 ± 19.27	198.12 ± 35.99	0.035	0.972
Bleeding volume during surgery (mL, mean ± SD)	116.10 ± 27.43	108.10 ± 32.37	1.133	0.186

**Table 3:** The incidence of anastomotic leakage, anastomotic stenosis, delayed gastric emptying, and pulmonary infection in different groups.

	Anastomotic leakage	Anastomotic stenosis	Delayed gastric emptying	Pulmonary infection
Observational Group (*n* = 50)	0	0	0	3
Control Group (*n* = 50)	1	2	0	1
χ^2^	0.154			
*P*	0.695			

The gastrointestinal symptom rating scores and the results of the six-minute walk test at discharge were compared between the two groups, as shown in Table 4. The six-minute walk distance in the observation group was significantly higher than that in the control group [(388.5 ± 26.8) m vs. (306.7 ± 40.6) m; *P* < 0.01]. The observation group had a significantly lower gastrointestinal symptom rating score compared with the control group; the difference was statistically significant (*P* < 0.01).

**Table 4:** The 6-minute walk test and PG-SGA score of the two groups at discharge.

Item	Control group (*n* = 50)	Observation group (*n* = 50)	*t*	*P*
6-minute walk test	306.7 ± 40.6	388.5 ± 26.8	11.9	0.00
GSRS	4.31 ± 1.33	2.78 ± 1.02	−6.46	0.00

The scores of quality of life (QLQ-C30) were compared between the two groups during the return visits to the hospital one month after surgery, as shown in Table 5. There was statistical significance in physical function, emotional function, and general health condition in the observation group (*P* < 0.01).

**Table 5:** Quality of life QLQ-C30 of two groups at return visit in the hospital one month after surgery.

		Control group (*n* = 50)	Observation group (*n* = 50)	*t*	*P*
Functional domain	Physical function	72.1 ± 12.8	79.6 ± 12.7	3.2	<0.01
	Role function	88.0 ± 12.6	91.0 ± 12.7	1.2	0.2
	Emotional function	50.8 ± 14.6	66.7 ± 21.8	4.3	<0.01
	Social Functions	86.3.7 ± 9.9	87.7 ± 13.8	0.6	0.58
	Cognitive function	80.3 ± 19.5	81.7 ± 13.6	−0.4	0.7
General health condition		57.8 ± 17.8	73.8 ± 16.4	4.7	<0.01

The scores of quality of life (QLQ-OES18) of patients with esophageal cancer one month after discharge were compared, as shown in Table 6. The observation group was lower than the control group in respect of dysphagia, eating, reflux, pain domains, and choking symptoms; the differences were statistically significant (*P* < 0.01), and there were no statistically significant differences in the other symptoms between the two groups (*P* > 0.05) (see Table 6).

**Table 6:** Comparison of scores of quality of life (QLQ-OES18) of patients with esophageal cancer in two groups at return visit in the hospital 1 month after surgery.

		Control group (*n* = 50)	Observation group (*n* = 50)	*t*	*P*
Domain	Dysphagia	38.0 ± 16.5	24.7 ± 14.1	−4.35	0.00
	Eating	31.1 ± 16.1	20.7 ± 9.5	−3.93	0.00
	Reflux	41.0 ± 16.2	23.3 ± 22.3	−4.53	0.00
	Pain	21.1 ± 14.8	13.6 ± 11.5	−2.85	0.005
Symptom	Saliva swallowing	22.0 ± 23.0	14.7 ± 16.7	−1.83	0.07
	Choking	40.7 ± 26.3	15.3 ± 20.4	−5.37	0.00
	Dry mouth	11.3 ± 19.8	8.0 ± 14.4	−0.97	0.34
	Decreased appetite	12.7 ± 16.3	14.0 ± 16.6	0.40	0.69
	Cough	23.3 ± 15.4	21.3 ± 16.2	0.63	0.52
	Speaking	24.7 ± 20.0	23.3 ± 15.4	−0.37	0.71

## Discussion

Due to the characteristic of progressive dysphagia, patients with esophageal cancer experience serious malnutrition resulting from long-term insufficient food intake. The surgical treatment methods for esophageal cancer are characterized by long operative times and substantial trauma, increased body consumption and decreased food intake, and further incidence of malnutrition (13), which affects various patient aspects, such as longer length of hospital stays, more complications, and increased medical expenses, thus leading to reduced quality of life. The guidelines for perioperative treatment of esophageal cancer recommended by the Enhanced Recovery After Surgery society state that early oral feeding has a positive effect in preventing vomiting, aspiration pneumonia, and other serious complications (14, 15). Therefore, implementing the early oral food intake is essential for postoperative patients with esophageal cancer.

### The Early Oral Food Intake Effectively Reduces the Gastrointestinal Symptoms of Postoperative Patients With Esophageal Cancer

Ha et al. (16) found that patients with esophageal cancer had different degrees of symptoms, even up to 15 symptoms, which may have been associated with the reconstruction of the digestive tract after resection of esophageal cancer. During resection, the normal anti-reflux structure was destroyed and the stomach was moved up to the chest, which caused the patients to have reflux, diarrhea, loss of appetite, and other symptoms. The recurrence of these gastrointestinal symptoms, plus concern about the treatment risk and prognosis, caused sadness, anxiety, and other negative emotions in patients (17), which affected their food intake and effect, aggravated their malnutrition, and further affected their quality of life and prognosis. This study showed that the gastrointestinal symptom rating score of the observation group was lower than that of the control group. Cause analysis is as follows: ① The observation group was able to continue oral feeding on the first day after surgery; this can promote intestinal tract movement, reduce bacterial translocation, enhance positive nitrogen equilibrium, and protect intestinal mucosal function, thus boosting the recovery of gastrointestinal function. ② Moreover, early feeding of patients proved safer when the fine food intake strategy was implemented under the guidance of medical staff, which reduced negative emotions of patients concerned about postoperative complications due to improper food intake. It also helped develop good intake habits and improved compliance. ③ The joint participation of family members in feeding management provided positive emotional support to patients, so that the patients were able to gradually adapt to the changes in digestive tract mode after surgery and form an effective response mechanism. Therefore, the early oral food intake strategy should be used for postoperative physiological and psychological interventions of patients with esophageal cancer, so the patients can incorporate these interventions into their own abilities. This strategy mobilizes the body’s positive emotions and improves its daily response capacity, thus reducing the occurrence of gastrointestinal symptoms.

### The Early Oral Food Intake Can Promote the Patients’ Rapid Recovery

This study showed that the six-minute walk distance in the observation group was significantly higher than that in the control group [(388.5 ± 26.8) m vs. (306.7 ± 40.6) m]; the difference was statistically significant (*P* < 0.01). Cause analysis is as follows: The patients in the control group required more intravenous nutrition support during fasting, so longer infusion time and more indwelt tubes made it difficult to implement early postoperative activities. The early oral food intake in the observation group reduced the time of gastric tube indwelling and liquid infusion and facilitated early activities of patients. Early activities not only helped maintain muscular function and prevented bed rest-related complications but also played a positive role in postoperative recovery (18). The six-minute walk test is an evaluation method that can comprehensively reflect the patients’ physical status and rehabilitation effect. The test results verified the positive effect of the early oral food intake strategy.

### The Early Oral Food Intake Strategy Can Improve the Patients’ Quality of Life

The results showed that the observation group was superior to the control group in respect of the quality of life scores, including physical function, emotional function, and general health condition one month after discharge. The scores in dysphagia, eating, reflux, pain domains, and choking symptoms in the observation group were lower than those in the control group. Cause analysis is as follows: In the observation group, the patients started eating on the first day after surgery and quantified and refined the types of food in each stage under the guidance of medical staff. The patients and family members had a better knowledge of food intake, so these patients had a higher compliance with intake management after discharge and a more comprehensive and balanced nutrition, which effectively diminished the concept that eating nothing or eating less would prevent postoperative complications. If no abnormality was found in the digestive tract angiography seven days after surgery, the patients in the control group were instructed to eat. If no discomfort was observed one to two days after food intake, the patients were discharged. After discharge, the patients’ food intake and types are limited, and the patients and their family members may develop a worse understanding and become uncompliant; food intake cannot meet the body’s needs, or they do not know how to respond to the discomfort symptoms. The patients in the observation group had decreased gastrointestinal symptoms and reduced physical and psychological burden, as well as indirectly improved quality of life. This was also demonstrated in a study by Zhang et al. (19) Wain et al. (20) discovered that successful postoperative recovery for esophageal cancer depended on the patients’ knowledge of how to eat again. In this study, the patients were instructed in implementing the early oral food intake strategy to quantify and refine food intake using detailed pictures, videos, and other materials. The strategy is simple and easy to learn. After patients learn to eat again, they can quickly adapt to the new eating method after reconstruction of the digestive tract and can have improved quality of life.

## Conclusion

In conclusion, early oral food intake meets the physiological needs of normal intake for patients after surgery. The fine food intake strategy provides a guarantee of intake safety, which reduces the occurrence of gastrointestinal symptoms, promotes a postoperative recovery effect, and improves postoperative quality of life, making it beneficial to clinical practice.

## Data Availability

The raw data supporting the conclusions of this article will be made available by the authors, without undue reservation.
